# Methods for evaluating innovative surgery: a nested ideal phase 2 study within an external randomised pilot (the ROMIO trial)

**DOI:** 10.1186/1745-6215-16-S2-O54

**Published:** 2015-11-16

**Authors:** Jane Blazeby, Richard Berrisford, Dan Titcomb, Andrew Hollowood, Grant Sanders, Christopher Streets, Tim Wheatley, Kerry Avery, George Hanna, Chris Metcalfe, Paul Barham

**Affiliations:** 1 University Hospitals Bristol NHS Foundation Trust, Bristol, UK; 2 University of Bristol, Bristol, UK; 3 Plymouth Hospitals NHS Trust, Plymouth, UK; 4 Imperial College London, London, UK

## Introduction

Pragmatic surgical RCTs are needed but it is unclear how to design studies with evolving innovative interventions. The aim of this paper was to describe how a pilot RCT comparing two standard techniques incorporated an additional randomised group with a nested IDEAL (Idea, Development, Evaluation, Assessment and Long-term evaluation of innovative surgery) Phase 2b evaluation of an evolving technique to inform main trial design.

## Methods

In centre one (three surgeons), patients were randomised to two types of standard surgery. In centre two (six surgeons), patients were also randomised to a third group including an evolving new surgical technique. Surgical protocols for standard techniques were agreed and monitored, whereas the evolving technique's protocol was flexible to allow innovations, yet outcomes and processes to be monitored. Patients were blinded to intervention received using large wound bandages.

## Results

Over 50 centre months, 237 patients were assessed for eligibility, 154 (65%) were eligible and 120 (78%) participated. Most (86%) received their randomised allocation. Patients were successfully blinded whilst assessing pain during the first week. In the evolving technique, surgery changed after three months, with a modified approach and anastomosis. This further evolved but did not stabilise during the pilot study. The main trial has been designed with a two-group comparison and the nested IDEAL study will continue in selected centres.

## Conclusion

This pilot RCT with a nested IDEAL phase 2 study including an innovative intervention shows that this method is feasible and informs the design of a main trial.

